# Multilayer hexagonal silicon forming in slit nanopore

**DOI:** 10.1038/srep14792

**Published:** 2015-10-05

**Authors:** Yezeng He, Hui Li, Yanwei Sui, Jiqiu Qi, Yanqing Wang, Zheng Chen, Jichen Dong, Xiongying Li

**Affiliations:** 1 School of Materials Science and Engineering, China University of Mining and Technology, Xuzhou, 221116, People’s Republic of China; 2 Key Laboratory for Liquid-Solid Structural Evolution and Processing of Materials, Ministry of Education, Shandong University, Jinan 250061, People’s Republic of China

## Abstract

The solidification of two-dimensional liquid silicon confined to a slit nanopore has been studied using molecular dynamics simulations. The results clearly show that the system undergoes an obvious transition from liquid to multilayer hexagonal film with the decrease of temperature, accompanied by dramatic change in potential energy, atomic volume, coordination number and lateral radial distribution function. During the cooling process, some hexagonal islands randomly appear in the liquid first, then grow up to grain nuclei, and finally connect together to form a complete polycrystalline film. Moreover, it is found that the quenching rate and slit size are of vital importance to the freezing structure of silicon film. The results also indicate that the slit nanopore induces the layering of liquid silicon, which further induces the slit size dependent solidification behavior of silicon film with different electrical properties.

In the last decades, low-dimensional materials have gained rapid development along with the microminiaturization trend of semiconductor industry. Graphene, an allotrope of carbon in the form of a two-dimensional (2D), one atom thick, hexagonal lattice, has always grabbed the worldwide attention ever since the experimental preparation in 2004^1^, due to its fascinating properties and nearly infinite applications^2^,^3^,^4^. Inspired by the tremendous advancement in graphene, other 2D materials begin to attract an increasingly scientific interest. Recently, strong effort has been invested to provide theoretical and experimental evidence for similar 2D materials composed of other group-IV element, such as silicon^5^,^6^, which is expected to have a great impact on the development of future nanoelectronic devices. The silicon analogue of graphene was first pointed out by Takeda and Shiraishi and was named silicene by Guzmán-Verri *et al.* in 2007^7^,^8^. It has been shown that the silicene with Si atoms packed in a buckled honeycomb lattice, is predicted to possess massless Dirac fermions and to exhibit a detectable quantum spin Hall effect, and other attractive properties^9^,^10^. Recent experiment obtained an estimated Fermi velocity for the silicene layer of *v*_*F*_ = 1.3 × 10^6^ ms^−1^ 11. Due to the specific physical and chemical properties, silicene has broad application prospects in electronic device, energy storage, and so on^12^,^13^. Currently, Li *et al.*^14^ reported a silicene field-effect transistor operating at room temperature, which opens up new opportunities for 2D silicon for various fundamental science studies and electronic applications.

Different from the carbon, the valence electrons of silicon localized in σ bonds are less mobile than those in graphite, which prevent the formation of extended π electronic states at low binding energies^15^. As a result, the silicon crystal tends to adopt the three-dimensional diamond structure made of sp3-hybridized Si atoms and there seem to be not any natural solid phase of silicon similar to graphite, which precludes the exfoliation method to generate silicene as performed initially in the case of graphene. Now, the most common way to prepare silicene is to deposit silicon on a silver substrate^16^,^17^,^18^. Although plenty of theoretical studies had speculated about the existence of silicene, the silicene was not reported experimentally until 2010, when the silicene nanoribbons on Ag(110) with graphene-like electronic signature was observed^19^,^20^. Thereafter, Daniele *et al.*^21^ systematically investigated structural and electronic properties of silicene nanosheets epitaxial grown on Ag(111) by combining scanning tunneling microscopy and scanning tunneling spectroscopy and evidence the presence of corrugated silicene domains.

Though a fair amount of efforts have been devoted to investigating the silicene, the preparation of high quality silicene films is still a major challenge in this field. Recently, some efforts have been devoted to studying the phase transition of confined silicon. Morishita *et al.*^22^,^23^ used molecular dynamics (MD) simulations to study the formation of nanowire, single- and double-layer silicon in slit pores and the stability is further confirmed by first principles MD calculations up to 300 K, which demonstrate the possibility of the synthesis of novel nanostructures by confinement in nanopores. Afterwards, the electronic and phonon properties of the double-layer silicon computed by Bai *et al.*^24^ suggested that the bilayer hexagonal silicon is a quasi-2D semimetal. In this paper, we perform molecular dynamics (MD) simulations to study the liquid-solid transition of silicon confined in a slit nanopore and provide evidence for the formation of multilayer silicene-like polymorph. The effect of cooling rate and slit size has been considered.

## Results

**Figure 1:**
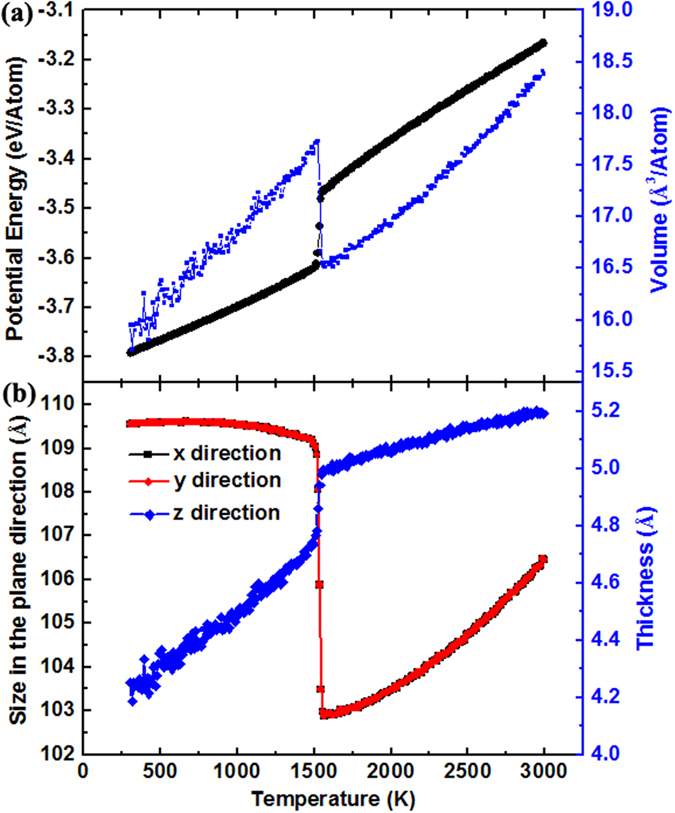
Freezing process of confined silicon films. (**a**) Potential energy and volume per atom as a function of temperature; (**b**) Size of silicon film in x, y and z directions at different temperature.

In this study, MD simulations are used to investigate the solidification of liquid silicon confined between two isolated and smooth walls. The liquid silicon is equilibrated at 3000 K first, followed by a cooling process to 300 K, at a quenching rate of 0.1 K/ps. Figure 1(a) shows the potential energy (PE) and volume per atom as a function of temperature. It can be seen that the PE and volume show a strong dependence on the temperature. At the beginning of freezing process, both the PE and the volume drop slowly with the decrease of temperature. At *T* = 1530 K, the PE suddenly drops by about 0.15 eV/atom from −3.465 eV/Atom to −3.615 eV/Atom. At the same time, the volume per atom increases sharply and the density of silicon film changes from 2.82 g/cm^3^ to 2.63 g/cm^3^. To clarify the reason of volume expansion, we plot the size of silicon film in x, y and z directions at different temperature in Fig. 1(b). It can be found that though the thickness of silicon film decreases at *T* = 1530 K, the plane (x, y) sizes of silicon film increase sharply, which induce the volume expansion of the system.

**Figure 2:**
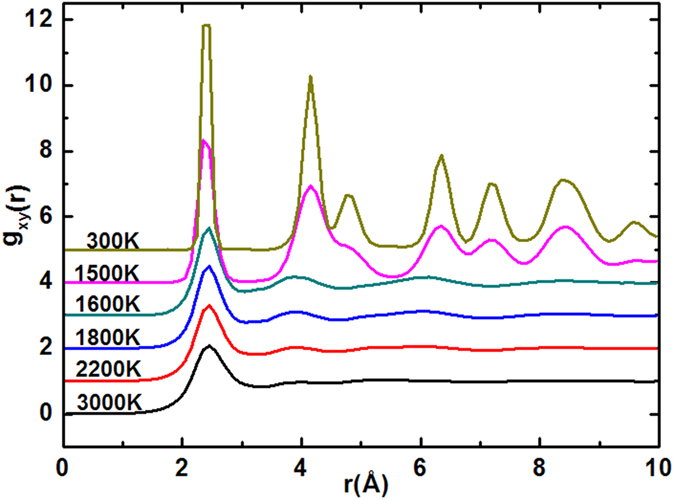
Lateral pair correlation function g_xy_(r) at different temperature.

Usually speaking, such a sudden change in PE and volume suggests the existence of a phase transition^25^,^26^,^27^. To better characterize the structural transition during the freezing process, we have plotted the lateral radial distribution functions *g*_*xy*_*(r)* at different temperature in Fig. 2. It is worth noting that the peak height of the *g*_*xy*_*(r)* increases significantly with the decreasing temperature, which indicates the structural transition from disordered state to ordered during the cooling process. When the temperature drops to 1500 K, obvious crystal peak begin to appear in the *g*_*xy*_*(r)*, suggesting that the liquid-solid phase transition of silicon film occurs and the system is transformed into ordered crystal structure. With the further decrease of temperature, the crystal peak becomes more obvious and the ordered degree of silicon film increases. In terms of the physical implication of *g*_*xy*_*(r)*, the abscissa value *r*_*i*_ of the *i*th peak always represents the average distance from *i*th nearest-neighbor shell to the central atom^28^. For example, the *r*_*1*_ corresponds to the average distance of the first nearest-neighbor shell. Another function of *g*_*xy*_*(r)* is that scaling behavior of *r*_*i*_/*r*_*1*_ should be the general feature for the liquid, glassy and crystalline states. At T = 300 K, the abscissa values of the first three peak are *r*_*1*_ = 2.4 Å, *r*_*2*_ = 4.15 Å and *r*_*3*_ = 4.75 Å. The ratios *r*_*2*_/*r*_*1*_ and *r*_*3*_/*r*_*1*_ happen to be about 1.73 and 1.98(approximately 2) respectively, which predicts the solidification structure of silicon film may be composed of hexagonal lattice.

**Figure 3:**
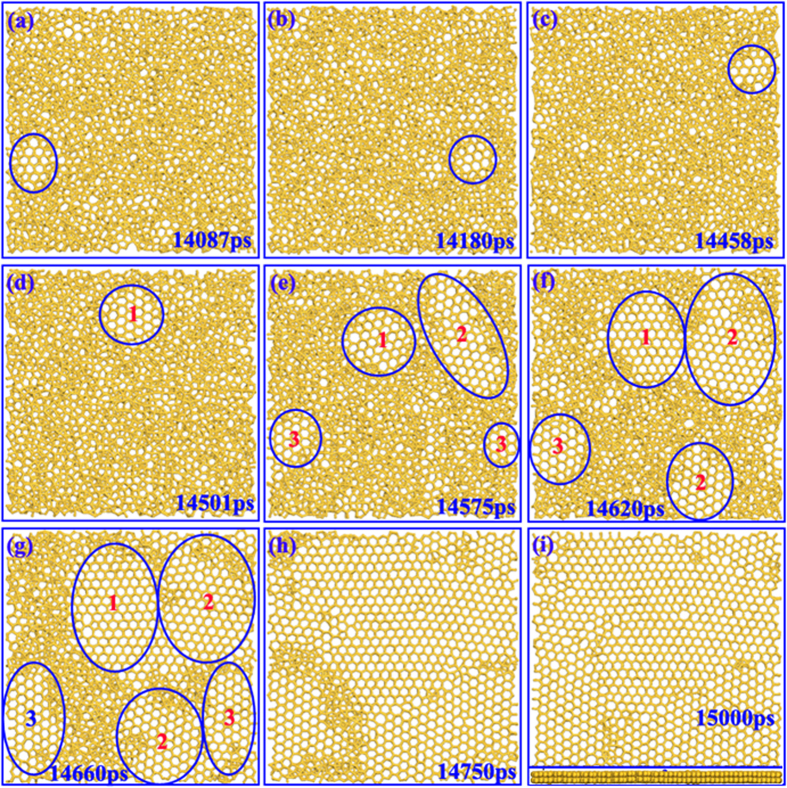
Structural evolution of liquid silicon in solidification process. (**a–c**) show the formation of silicon islands while (**d–i**) show the crystal nucleation and growth.

To explore the microscopic details of the structure evolution during the freezing process, simulation snapshots are displayed in Fig. 3. It can be found that the structure has been dramatically transformed during the cooling process. As shown in Fig. 3(a–c), random silicon island appears in the liquid silicon before the liquid-solid phase transition. However, it is worth noting that the silicon islands in this stage can persist only for a short time and are easy to break into disordered structure inversely. If the size of islands exceeds a critical value, the islands can stable exist as a nucleus as shown in Fig. 3(d), which indicates the beginning of liquid-solid phase transition. With the further decrease of temperature, the liquid silicon is simultaneously nucleated at individual islands. Subsequently, the islands begin to grow outwards by incorporating the disordered structure. Then, the carbon islands will encounter each other and combine to form a complete polycrystalline film. Because the lattice orientation of every island is quite different, there may be some grain boundaries in the solidification structure. Eventually, the 2D silicon exhibits a bilayer hexagonal polycrystalline structure as shown in Fig. 3(i), agrees well with previous results^24^.

**Figure 4:**
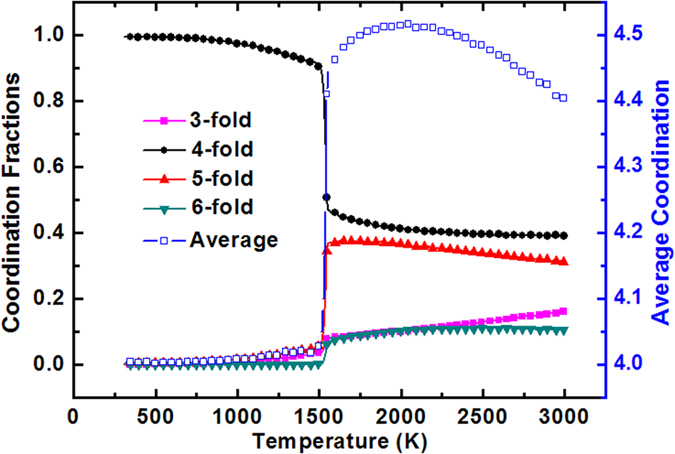
Fractions of atoms with different coordination number and average coordination as a function of temperature.

The phase transition can also be indicated by a sudden change in coordination number. In this study, the coordination of each atom counts neighbors within a radius of 3 Å. As shown in Fig. 4, it can be found that the coordination fractions are strongly dependent on the slit size. Before solidification, the average coordination first increases and then decrease slowly with the decreasing temperature. In the stage of liquid-solid phase transition, the average coordination varies dramatically from 4.45 to 4.02. At this point, the fraction of 4-fold coordination increases sharply while the fraction of the other coordination types decreases steeply. At *T* = 300 K, the fraction of 4-fold coordination reaches nearly 100 percent, rather different from the 3-fold coordination dominated graphite, although the silicon film and graphite both have hexagonal lattice. This is because the silicon atoms between two layers are combined by σ bonds rather than the weaker π bonds^15^.

**Figure 5:**
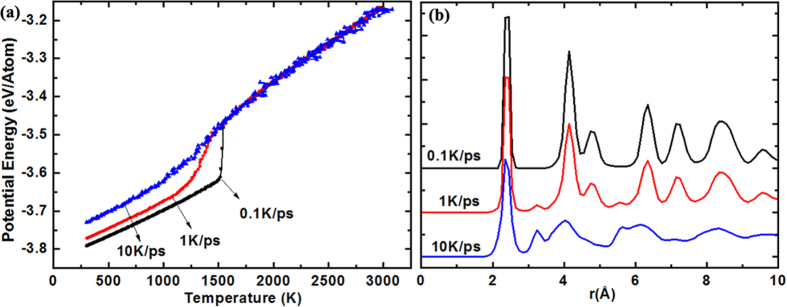
Freezing process at different quenching rates. (**a**) Potential energy per atom versus temperature (**b**) Lateral pair correlation function of silicon films (300 K).

Next, we will discuss the freezing process of silicon film at three different quenching rates (QRs). As shown in Fig. 5(a), the QR has little effect on the change of PE before solidification. With the decreasing temperature, there is a sharp decline in PE curve, indicating the liquid-solid phase transition. It is worth noting that the decline ranges of PE curves decrease and are getting less stark as the QR increases. At QR = 0.1 K/ps, the PE suddenly drops at *T* = 1530 K, indicating the completion of solidification at this temperature. However, the PE curve does not show a dramatic decline in a certain temperature but in a range of temperature when the QRs are 1 K/ps and 10 K/ps, usually indicating the generation of amorphous structure. To further discriminate the solidification structure, the *g*_*xy*_*(r)* of solidification structure at different QRs are plotted in Fig. 5(b). It can be found that the crystal peak of *g*_*xy*_*(r)* at QR = 0.1 K/ps is very obvious, suggesting that the system is dominated by ordered structure. At QR = 10 K/ps, the second peak of *g*_*xy*_*(r)* splits into two subpeaks and there is no crystal peaks, which indicates that the solidification structure is amorphous. At QR = 1 K/ps, the crystal peak and the splitting second peak exits in the *g*_*xy*_*(r)* at the same time, indicating the coexistence of ordered and disordered structures.

**Figure 6:**
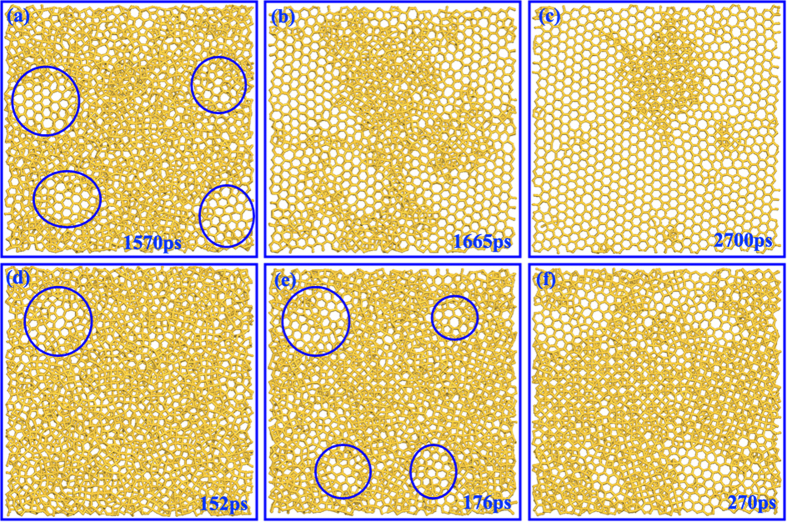
Structural evolution at different quenching rates. (**a**–**c**) show the solidification process at QR = 1 K/ps while (**d–f**) show the solidification process at QR = 10 K/ps.

To explore the microscopic details of the structure evolution at different QRs, Fig. 6 shows the simulation snapshots at different QRs. As shown in Fig. 6(a), the nucleation and growth of liquid silicon begins with four stable islands at QR = 1 K/ps. However, due to the fast cooling process, the crystal nuclei do not have enough time to grow up and a part of liquid silicon directly freezes into amorphous structure before converting to ordered structure, which induces the coexistence of ordered and disordered structures. This phenomenon becomes even more clear at QR = 10 K/ps. As shown in Fig. 6(d–f), there are only a few silicon around the nucleation island transformed into ordered structure and most of liquid silicon directly freezes into amorphous structure. As a result, the *g*_*xy*_*(r)* shows the amorphous characteristics.

**Figure 7:**
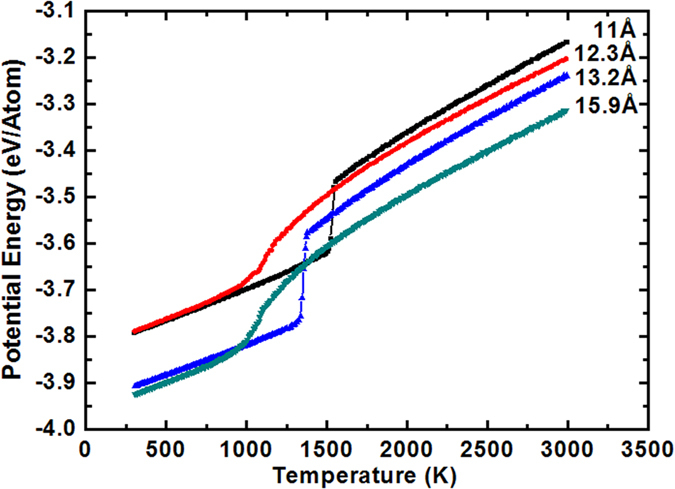
Potential energy per atom versus temperature for different slit sizes.

As clarified above, the QR is of vital importance to the freezing structure of silicon film. Next, we will investigate how the slit size affects the solidification behavior of the liquid silicon at QR = 0.1 K/ps. Figure 7 shows the PE per atom as a function of temperature for different silt sizes *D*. It is can be found that there is obvious sudden drop in the PE curves at *T* = 1530 K and 1360 K for *D* = 11 Å and 13.2 Å respectively, suggesting that the silicon films solidify at that temperature and is dominated by the ordered structure. It is also worth noting that the freezing point of silicon shifts towards the lower temperature when *D* changes from 11 Å to 13.2 Å. For D = 12.3 Å and 15.9 Å, the PE curve does not show a dramatic decline in a certain temperature but in a range of temperature, indicating the generation of amorphous structure.

**Figure 8:**
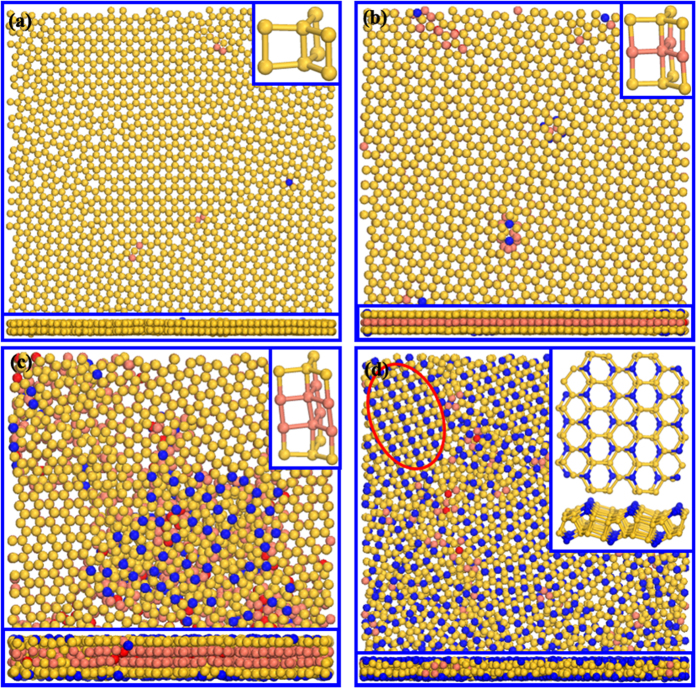
Solidification structure for different slit sizes. Blue: 3-fold coordinated atoms; Yellow: 4-fold coordinated atoms; Brown: 5-fold coordinated atoms; Red: other atoms.

To further study the microscopic structure of silicon film after solidification, simulation snapshots at 300 K are investigated, as displayed in Fig. 8. It can be found that the solidification structure is very different for different slit sizes. The silicon film for *D* = 11 Å and 13.2 Å is composed of two and three atomic layers with hexagonal lattice, respectively. At *D* = 11 Å, the atoms in the two layers are mainly 4-fold coordinated. At *D* = 13.2 Å, the atoms in the outer layers are mainly 4-fold coordinated, while the atoms in the inner layers are mainly 5-fold coordinated, so that the whole film is a 4-5-4 coordinated sandwich structure. At *D* = 15.9 Å, the silicon films is composed of both ordered hexagonal structure and disordered structure. Here, the hexagonal structure is constructed by four atom layers coordinated as 4-5-5-4. Different form the case at *D* = 15.9 Å, though the silicon films also contains both ordered hexagonal structure and disordered structure at *D* = 12.3 Å, the hexagonal structure is not plane but buckled, as shown in the inset of Fig. 8(d). The buckled hexagonal structure is induced by the protuberance of one 3-fold coordinated atom in every ring. The protruding atom lines arrange alternatively in the two layers, which induces the fluctuation of silicon film.

**Figure 9:**
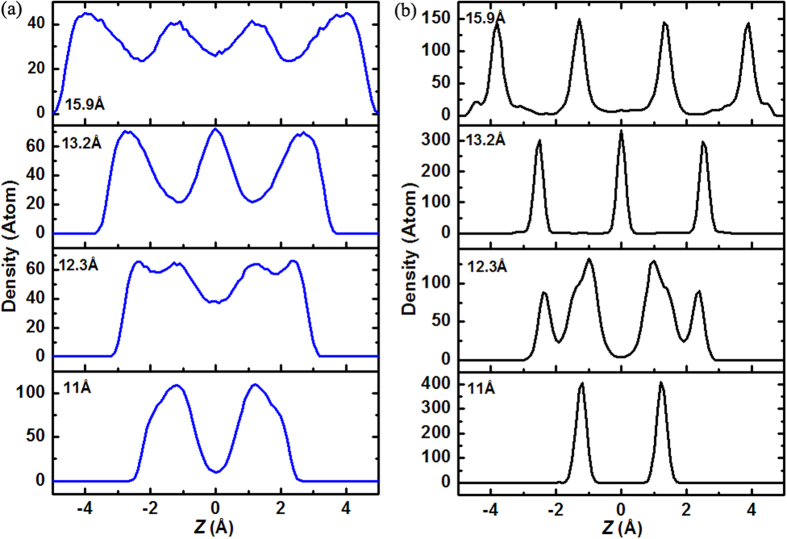
Density distribution functions along the confined direction of the liquid and solid silicon for different slit sizes. (**a**) 1800 K (liquid); (**b**) 300 K(solid).

In order to clarify the reasons for such the slit size dependent solidification behavior of silicon film, we have plotted the density distribution functions along the confined direction for the liquid (1800 K) and solid (300 K) silicon in Fig. 9. The results clearly suggest a structural correlation between the liquid and solid silicon. At D = 11 Å, 13.2 Å and 15.9 Å, the density distribution functions of the liquid and solid silicon both have two, three and four peaks as shown Fig. 9, indicating the two phases are both composed of two, three and four atomic layers, although the density peaks of liquid silicon are rather smooth than that of the solid one. At D = 12.3 Å, each main density peak of the liquid silicon split into two subpeaks, which becomes more obvious in the solid silicon corresponding to the buckled hexagonal structure. Based on the above, one can draw a conclusion that the structural difference of silicon film has already appeared before solidification and the layer number is determined by the slit size. With the decreasing temperature, the layering phenomenon is increasingly evident. This result is in good agreement with previous experimental and numerical studies that confined liquid undergoes a layering transition nearing the solid wall^29^,^30^,^31^. Therefore, it can be deduce that the slit nanopore induces the layering of liquid silicon, which further induces the slit size dependent solidification behavior of silicon film.

**Figure 10:**
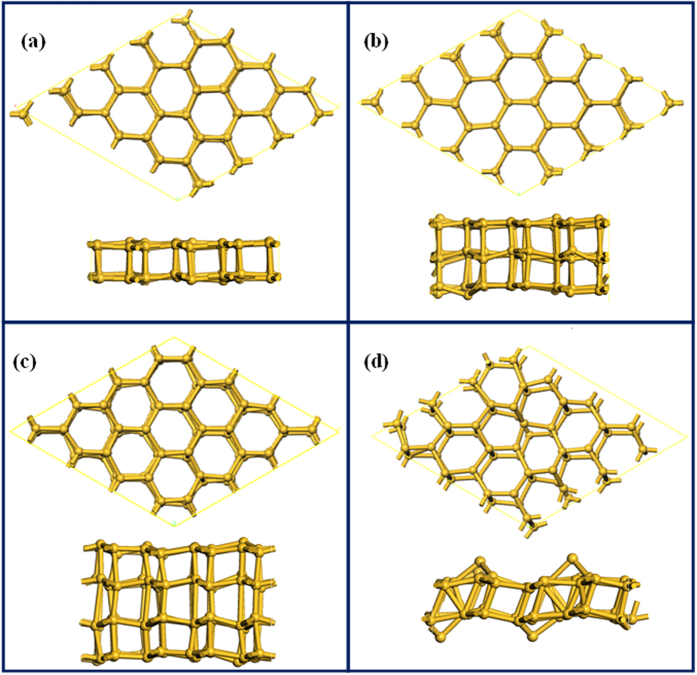
Top and side views of multi-layer silicon at 400 K examined by first-principles MD calculations. (**a**) 2-layer silicon; (**b**) 3-layer silicon; (**c**) 4-layer silicon; (**d**) Buckled 2-layer silicon.

**Figure 11:**
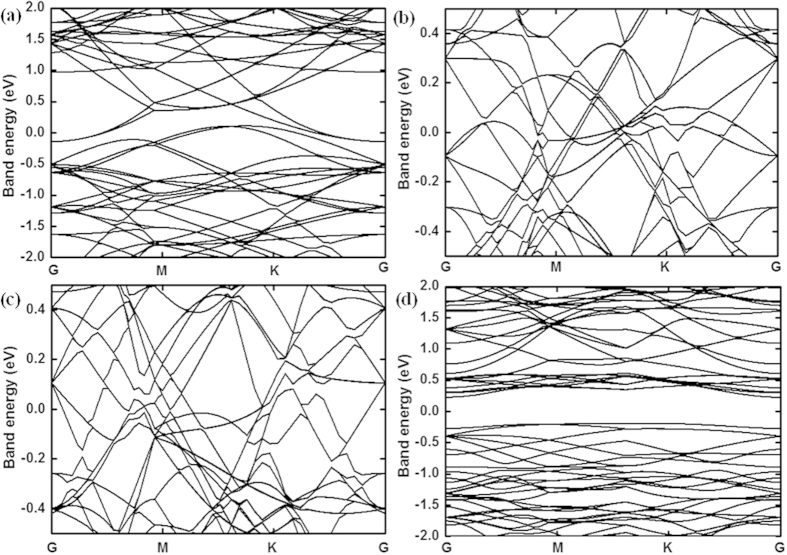
Band energy of multi-layer silicon. (**a**) 2-layer silicon; (**b**) 3-layer silicon; (**c**) 4-layer silicon; (**d**) Buckled 2-layer silicon.

The stability of stand-alone multi-layer silicon (without the confinement) has been examined by first-principles MD calculations. The temperature and pressure of 2-layer (64 atoms), 3-layer (96 atoms) and 4-layer (128 atoms) structures are set to be 400 K and zero respectively, using the Nosé-Andersen method. The time step is 1fs. Figure 10 shows the snapshots of the first-principles MD simulation at 10 ps. It is found that there is no obvious structural transition except for slight vibrations of silicon atoms, which indicates that the stand-alone bilayer silicon can still stably persist at 400 K. After confirming the stability of the multi-layer hexagonal silicon polymorph, the electronic properties are also explored by first-principles MD calculations. The Brillouin zone was sampled with (4 × 4 × 1) k points of a Monkhorst–Pack grid. Figure 11 shows the band structures of multi-layer hexagonal silicon after full geometry optimization. It can be found that the 2-layer silicon shows a classic semimetal characteristic. With the increase of layer number, the silicon tends to become a metal, as shown in Fig. 11(c). Figure 11(d) shows that the buckled 2-layer silicon is semi-conductor of electricity.

## Discussion

MD simulations have been performed to study the cooling process of liquid silicon confined in slit nanopore. With the decrease of temperature, the silicon island randomly appears in the liquid first and then grows up to grain nucleus, indicating the start of freezing transition. Subsequently, the liquid silicon transforms to multilayered hexagonal film in a short period, along with dramatic change in potential energy, atomic volume, coordination number and lateral radial distribution function. For the ordered hexagonal structure, the atoms in the layer adjacent to the wall are mainly 4-fold coordinated while inner layer are dominated by 5-fold coordinated atoms, which is rather different from the multilayer graphene.

The quenching rate and slit size are both very important to the solidification behavior of silicon film. On the one hand, the increasing quenching rate will lead to the non-crystallizing of the liquid silicon. On the other hand, the increasing slit size not only affects the structure of each layer, but also introduces new layers in the multilayer film. Our results clearly show that the slit nanopore induces the layering of liquid silicon, which further induces the slit size dependent solidification behavior of silicon film. First-principles MD calculations are used to confirm the structural stability of multi-layer silicon. The band energy calculations suggest that layered silicon with different structures has different electrical properties. These findings provide physical and dynamic insights into the liquid-solid phase transition in 2D silicon and predict a possible way to fabricate the 2D silicon materials with different properties.

## Methods

In this study, MD simulations are performed to investigate the solidification of liquid silicon confined between two isolated and smooth walls. The Stillinger-Weber empirical potential is employed to describe the silicon-silicon interaction^32^, which comprises two and three body terms, energetically favoring tetrahedral coordination of the atoms. We utilize the 12-6 Lennard-Jones (LJ) potential, with a well depth ε = 0.310 kcal/mol and size parameter σ = 3.804 Å^33^,^34^,^35^, to describe the silicon-wall interaction. Although the SW potential has known limitations^36^, it provides a reasonable description of the crystalline and liquid phase^37^,^38^,^39^ and has been widely used to study the structure transition of silicon^40^,^41^,^42^.

For all simulations, we adopt the constant number, lateral-pressure, temperature (NPT) ensemble and use the velocity-verlet algorithm with an integration time step of 1 fs. In this work, we use the MD package LAMMPS to perform the simulations^43^. The periodic boundary conditions are applied only in the lateral directions parallel to the wall at zero pressure, while in the vertical direction perpendicular to the wall, the slit size D between two walls is originally set to be 11 Å. For each D, the liquid silicon is equilibrated at 3000 K first, followed by a cooling process to 300 K, at a quenching rate of 0.1 K/ps. Then, the final structures are obtained after a relaxation process for 200 ps at 300 K. To confirm the structural stability and study the electrical property of multi-layer silicon, first-principles MD calculations are used based on density functional theory within the generalized-gradient approximation (GGA)^44^ with the Perdew-Burke-Ernzerhof (PBE) functional and ultrasoft pseudopotential^45^.

## Additional Information

**How to cite this article**: He, Y. *et al.* Multilayer hexagonal silicon forming in slit nanopore. *Sci. Rep.* **5**, 14792; doi: 10.1038/srep14792 (2015).

## References

[b1] Novoselov K. S. *et al.* Electric field effect in atomically thin carbon films. Science 306, 666–669 (2004). 15499015 10.1126/science.1102896

[b2] Novoselov K. S. *et al.* Two-dimensional gas of massless Dirac fermions in graphene. Nature 438, 197–200 (2005). 16281030 10.1038/nature04233

[b3] Kim K. S. *et al.* Large-scale pattern growth of graphene films for stretchable transparent electrodes. Nature 457, 706–710 (2009). 19145232 10.1038/nature07719

[b4] Seol J. H. *et al.* Two-Dimensional Phonon Transport in Supported Graphene. Science 328, 213–216 (2010). 20378814 10.1126/science.1184014

[b5] Feng B. *et al.* Evidence of Silicene in Honeycomb Structures of Silicon on Ag(111). Nano Lett. 12, 3507–3511 (2012). 22658061 10.1021/nl301047g

[b6] Lalmi B. *et al.* Epitaxial growth of a silicene sheet. Appl. Phys. Lett. 97, 223109 (2010).

[b7] Takeda K. & Shiraishi K. Theoretical possibility of stage corrugation in Si and Ge analogs of graphite. Phys. Rev. B 50, 14916–14922 (1994). 10.1103/physrevb.50.14916 9975837

[b8] Guzmán-Verri G. G. & Lew Yan Voon L. C. Electronic structure of silicon-based nanostructures. Phys. Rev. B 76, 075131 (2007).

[b9] Cahangirov S., Topsakal M., Akturk E., Sahin H. & Ciraci S. Two- and One-Dimensional Honeycomb Structures of Silicon and Germanium. Phys. Rev. Lett. 102, 236804 (2009). 19658958 10.1103/PhysRevLett.102.236804

[b10] Liu C.-C., Feng W. & Yao Y. Quantum spin Hall effect in silicene and two-dimensional germanium. Phys. Rev. Lett. 107, 076802 (2011). 21902414 10.1103/PhysRevLett.107.076802

[b11] Vogt P. *et al.* Silicene: Compelling Experimental Evidence for Graphenelike Two-Dimensional Silicon Lay. Phys. Rev. Lett. 108, 155501 (2012). 22587265 10.1103/PhysRevLett.108.155501

[b12] Tsai W. F. *et al.* Gated silicene as a tunable source of nearly 100% spin-polarized electrons, Nat. Commun. 4, 1500 (2012). 23422668 10.1038/ncomms2525

[b13] Tritsaris G. A., Kaxiras E., Meng S. & Wang E. Adsorption and Diffusion of Lithium on Layered Silicon for Li-Ion Storage. Nano Lett. 13, 2258–2263 (2013). 23611247 10.1021/nl400830u

[b14] Tao L. *et al.* Silicene field-effect transistors operating at room temperature. Nat. Nanotech. 10, 227–231 (2015). 10.1038/nnano.2014.325 25643256

[b15] Yamada-Takamura, Y. & Friedlein R. Progress in the materials science of silicene. Sci. Technol. Adv. Mater. 15, 064404 (2014). 10.1088/1468-6996/15/6/064404 PMC5090386 27877727

[b16] Lin C. L. *et al.* Structure of Silicene Grown on Ag(111). Appl. Phys. Express 5, 045802 (2012).

[b17] Jamgotchian H. *et al.* Growth of silicene layers on Ag(111): unexpected effect of the substrate temperature. J. Phys.: Condens. Matter. 24, 172001 (2012). 22487603 10.1088/0953-8984/24/17/172001

[b18] Scalise E. *et al.* Vibrational properties of epitaxial silicene layers on (111) Ag. Appl. Surf. Sci. 291, 113–117 (2014).

[b19] Aufray B. *et al.* Graphene-like silicon nanoribbons on Ag(110): A possible formation of silicene. Appl. Phys. Lett. 96, 183102 (2010).

[b20] De Padova P. *et al.* Evidence of graphene-like electronic signature in silicene nanoribbons. Appl. Phys. Lett. 96, 261905 (2010).

[b21] Chiappe D., Grazianetti C., Tallarida G., Fanciulli, M. & Molle A. Local Electronic Properties of Corrugated Silicene Phases. Adv. Mater. 24, 5088–5093 (2012). 22847791 10.1002/adma.201202100

[b22] Morishita T., Nishio K. & Mikami M. Formation of single- and double-layer silicon in slit pores. Phys. Rev. B 77, 081401(R) (2008).

[b23] Nishio K., Morishita T., Shinoda W. & Mikami M. Molecular dynamics simulations of self-organized polyicosahedral Si nanowire. J. Chem. Phys. 125, 074712 (2006). 16942369 10.1063/1.2337291

[b24] Bai J., Tanaka H. & Zeng X. C. Graphene-Like Bilayer Hexagonal Silicon Polymorph. Nano Res. 3, 694–700 (2010).

[b25] Han S., Choi M. Y., Kumar, P. & Stanley H. E. Phase transitions in confined water nanofilms. Nature Phys. 6, 685–689 (2010).

[b26] He Y. Z., Li H., Jiang Y. Y., Li X. Y. & Bian X. F. Liquid-liquid phase transition and structure inheritance in carbon films. Sci. Rep. 4, 3635 (2014). 24407276 10.1038/srep03635 PMC3887372

[b27] He Y. Z. *et al.* Atomic insight into copper nanostructures nucleation on bending graphene. Phys. Chem. Chem. Phys. 15, 9163–9169 (2013). 23649309 10.1039/c3cp50876e

[b28] Jiang Y. Y. *et al.* Anomalies in rapid cooling behavior of the Fe melts in nanoconfinement. EPL 97, 16002 (2012).

[b29] Granick S. Confined liquid controversies near closure? Physics 3, 73 (2010).

[b30] Li T. D., Gao J. P., Szoszkiewicz R., Landman U. & Riedo E. Structured and viscous water in subnanometer gaps. Phys. Rev. B 75, 115415 (2007).

[b31] Choi W. Y., Kang J. W. & Hwang H. J. Structures of ultrathin copper nanowires encapsulated in carbon nanotubes. Phys. Rev. B 68, 193405 (2003).

[b32] Stillinger F. H. & Weber T. A. Computer simulation of local order in condensed phases of silicon. Phys. Rev. B 31, 5262–5271 (1985). 10.1103/physrevb.31.5262 9936488

[b33] Lithoxoos G. P., Samios J. & Carissan Y. Investigation of silicon model nanotubes as potential candidate nanomaterials for efficient hydrogen storage: a combined ab initio/grand canonical Monte Carlo simulation study. J. Phys. Chem. C 112, 16725–16728 (2008).

[b34] Haeri H. H., Ketabi S. & Hashemianzadeh S. M. The solvation study of carbon, silicon and their mixed nanotubes in water solution. J. Mol. Model. 18, 3379–3388 (2012). 22271095 10.1007/s00894-011-1339-2

[b35] He Y. Z., Li X. Y., Li H., Jiang Y. Y. & Bian X. F. Layering transition in confined silicon. Nanoscale 6, 4217–4224 (2014). 24609530 10.1039/c3nr06174d

[b36] Buehler M. J., Tang H., van Duin A. C. T. & Goddard W. A. III Threshold Crack Speed Controls Dynamical Fracture of Silicon Single Crystals. Phys. Rev. Lett. 99, 165502 (2007) 17995264 10.1103/PhysRevLett.99.165502

[b37] Balamane H., Halicioglu T. & Tiller W. Comparative study of silicon empirical interatomic potentials. Phys. Rev. B 46, 2250 (1992). 10.1103/physrevb.46.2250 10003901

[b38] Broughton J. & Li X. P. Phase diagram of silicon by molecular dynamics. Phys. Rev. B 35, 9120 (1987). 10.1103/physrevb.35.9120 9941309

[b39] Cook S. J. & Clancy P. Comparison of semi-empirical potential functions for silicon and germanium, Phys. Rev. B 47, 7686 (1993). 10.1103/physrevb.47.7686 10004775

[b40] Sastry S. & Angell C. A. Liquid-liquid phase transition in supercooled silicon. Nature Mater. 2, 739–743 (2003). 14556000 10.1038/nmat994

[b41] Ganesh P. & Widom M. Liquid-Liquid Transition in Supercooled Silicon Determined by First-Principles Simulation. Phys. Rev. Lett. 102, 075701 (2009). 19257690 10.1103/PhysRevLett.102.075701

[b42] Vasisht V. V., Saw S. & Sastry S. Liquid-liquid critical point in supercooled silicon, Nature Phys. 7, 549–553 (2011).

[b43] Plimpton S. Fast Parallel Algorithms for Short-Range Molecular Dynamics, J. Comput. Phys. 117, 1–19 (1995).

[b44] Perdew J. P., Burke K. & Ernzerhof M. Generalized gradient approximation made simple. Phys. Rev. Lett. 77, 3865–3868 (1996). 10062328 10.1103/PhysRevLett.77.3865

[b45] Vanderbilt D. Soft self-consistent pseudopotentials in a generalized eigenvalue formalism. Phys. Rev. B 41, 7892–7895 (1990). 10.1103/physrevb.41.7892 9993096

